# Experimental and clinical usefulness of crossmodal paradigms in psychiatry: an illustration from emotional processing in alcohol-dependence

**DOI:** 10.3389/fnhum.2013.00394

**Published:** 2013-07-25

**Authors:** Pierre Maurage, Salvatore Campanella

**Affiliations:** ^1^Laboratory for Experimental Psychopathology, Faculty of Psychology, Institute of Psychology, Université Catholique de LouvainLouvain-la-Neuve, Belgium; ^2^Laboratory of Psychological Medicine and Addictology, ULB Neuroscience Institute, Université Libre de BruxellesBrussels, Belgium

**Keywords:** crossmodal, emotion, alcohol-dependence, social cognition, face, voice

## Abstract

Crossmodal processing (i.e., the construction of a unified representation stemming from distinct sensorial modalities inputs) constitutes a crucial ability in humans' everyday life. It has been extensively explored at cognitive and cerebral levels during the last decade among healthy controls. Paradoxically however, and while difficulties to perform this integrative process have been suggested in a large range of psychopathological states (e.g., schizophrenia and autism), these crossmodal paradigms have been very rarely used in the exploration of psychiatric populations. The main aim of the present paper is thus to underline the experimental and clinical usefulness of exploring crossmodal processes in psychiatry. We will illustrate this proposal by means of the recent data obtained in the crossmodal exploration of emotional alterations in alcohol-dependence. Indeed, emotional decoding impairments might have a role in the development and maintenance of alcohol-dependence, and have been extensively investigated by means of experiments using separated visual or auditory stimulations. Besides these unimodal explorations, we have recently conducted several studies using audio-visual crossmodal paradigms, which has allowed us to improve the ecological validity of the unimodal experimental designs and to offer new insights on the emotional alterations among alcohol-dependent individuals. We will show how these preliminary results can be extended to develop a coherent and ambitious research program using crossmodal designs in various psychiatric populations and sensory modalities. We will finally end the paper by underlining the various potential clinical applications and the fundamental implications that can be raised by this emerging project.

## Introduction

Crossmodal processing can be globally defined as the ability to build a unitary representation of one's environment on the basis of stimulations coming from different sensorial modalities (Driver and Spence, 2000). In everyday life, human beings are confronted with a constant flow of multi-sensorial stimulations, and the capacity to integrate them is thus crucial for daily adaptive behaviors like social interactions, spatial attention or perceptuo-motor coordination (Lalanne and Lorenceau, 2004; Campanella and Belin, 2007). The importance of these crossmodal processes has led many researchers to investigate their behavioral and cerebral correlates, and the exploration of crossmodal mechanisms now constitutes a flourishing field in cognitive psychology and neuroscience (Calvert et al., 2001; De Gelder and Bertelson, 2003; Amedi et al., 2005). Indeed, hundreds of studies have been conducted during the last two decades on crossmodality among healthy participants, and huge advances have undeniably been made in understanding the developmental, psychological and cerebral correlates of crossmodality (particularly of face-voice integration). A wide variety of multimodal tasks have been used, requiring the integration of gender, identity (e.g., Joassin et al., 2011a,b; Love et al., 2011) or emotional features (e.g., Dolan et al., 2001; Pourtois et al., 2005; Ethofer et al., 2006a,b, 2013; Kreifelts et al., 2007, 2009, 2010; Robins et al., 2009; Müller et al., 2011), and these various results led to the identification of several brain areas specifically involved in the multisensory integration (mainly the superior parietal lobule, inferior occipital, middle frontal and superior temporal sulci).

### Crossmodal processes in psychiatry: a serious lack of data

The exploration of crossmodality in healthy populations has obviously gained a central position in the experimental psychology and neuroscience domains and has come to maturity, as notably illustrated by the development of integrative models structuring the numerous experimental data available (e.g., Campanella and Belin, 2007 for a review). However, there is a patent contrast between the large number of studies exploring the efficient crossmodal processing and the paucity of data currently available concerning the impairment of this processing in pathological populations. Indeed, while scientific knowledge is classically enriched by results obtained from impaired populations, very few clinical crossmodal research projects have been conducted up to now. Crossmodal integration impairments have been explored in several populations presenting perceptual alterations (e.g., visual or auditory loss, Zupan and Sussman, 2009; Barone, 2010; Massida et al., 2011), but very few studies have focused on crossmodal processing in neurological and psychiatric populations. Among these studies, main results concerned schizophrenia (De Gelder et al., 2005; Pearl et al., 2009; Szycik et al., 2009; Seubert et al., 2010a; Van den Stock et al., 2011), autism (van der Smagt et al., 2007; Mongillo et al., 2008; Foss-Feig et al., 2010; Kwakye et al., 2011) and Alzheimer's disease (Delbeuck et al., 2007), and suggested large-scale crossmodal deficits in these populations, while unimodal processes may be preserved (e.g., De Jong et al., 2009). As these psychopathological states have been described as disconnection syndromes (Friston and Frith, 1995; Melillo and Leisman, 2009), these crossmodal impairments might be related to an alteration of binding abilities needed to connect and integrate the different sensorial inputs. Moreover, as crossmodal integration is crucial in daily life, these alterations could be at least partly responsible for cognitive and social alterations observed in psychiatric states. Up to now however, as only scarce data are available in schizophrenia and autism, and as crossmodality has not been explored in other psychiatric states, many questions have remained unexplored concerning the crossmodal processing in psychiatry.

This striking paradox between the extensive knowledge concerning “normal” crossmodal processing, notably for the integration of emotional stimuli (e.g., De Gelder et al., 1999; Pourtois et al., 2000, 2002; Chen et al., 2010; Kreifelts et al., 2010; Regenbogen et al., 2012; Klasen et al., 2012; Ethofer et al., 2013) and the very few data on impaired crossmodal integration in clinical populations thus constitutes a strong limit of the current knowledge on crossmodality, and voices recently rose to promote the development of crossmodal research among clinical populations, with a double aim. On the one hand, there is a need to obtain a better description of the impairments associated with pathological states, particularly by offering a more ecological and complete evaluation of the deficits. On the other hand, a renewal of the understanding of crossmodal integration among healthy subjects is urgently needed. Indeed, if behavioral deficits in crossmodal processing are observed in a clinical population, comparing the cerebral activations between this population and healthy controls will offer strong insights concerning the brain regions associated with crossmodal processing. As summarized by Laurienti et al. (2005), “the use of clinical populations can add to the battery of study designs available to the imaging scientist investigating multisensory integration.” Despite the great promise of this perspective, very little research has up to now attempted to improve our understanding of both pathological states and the mechanisms of multisensory integration in general through crossmodal research in clinical populations.

### Underlining the usefulness of crossmodal research in psychiatry

In view of the present limitations related to crossmodal research in psychiatric populations, the central objective of the present paper is to underline the usefulness of exploring crossmodal processing among clinical populations, and to help prepare the ground for the expansion of this innovative research topic. First, our recent studies exploring emotional crossmodal processing in alcohol-dependence will be described in order to illustrate the possibilities offered by this research field. Indeed, emotional decoding impairments have been found to be involved in the relapse after detoxification (e.g., Kornreich et al., 2001; Zywiak et al., 2003), and have been extensively investigated using visual or auditory stimulation (e.g., Monnot et al., 2001; Townshend and Duka, 2003, respectively). Capitalizing on these unimodal explorations, our research group has recently conducted several studies using audio-visual bimodal paradigms to increase the ecological validity of the experimental designs. The complementary use of behavioral, electrophysiological and neuroimaging techniques allowed obtaining the first insights concerning multimodal integration in alcohol-dependence. Second, these initial results will be discussed to show how they can (together with preliminary results obtained for multimodal integration in other psychiatric states) be extended to develop a coherent and ambitious research program using various psychiatric populations and sensory modalities. Finally, a conclusive section will underline the various potential clinical applications and the fundamental implications that this emerging project could bring.

## Emotional deficits in alcohol-dependence

### Alcohol-dependence: a serious mental health problem

Alcohol-dependence is the most frequent psychiatric diagnosis worldwide and is listed among the three more detrimental health problems (Harper and Matsumoto, 2005). Around 10% of the adult population in Western countries fulfils the diagnosis criteria for alcohol-dependence, and excessive alcohol consumption directly leads to 2.5 million deaths per year worldwide (World Health Organization, 2011). In view of these large-scale deleterious consequences of alcohol-dependence, extensive efforts have been conducted during the last decades to obtain a better understanding of alcohol-dependence at clinical and fundamental levels, particularly concerning the physiological, behavioral and cerebral impairments associated with chronic excessive alcohol consumption. Alcohol-dependence is known to have deleterious effects on many body systems (e.g., hepatic, cardio-vascular or gastrointestinal), but also on the central nervous system (see Oscar-Berman and Marinkovic, 2007 for a review,). Indeed, it has been extensively established that alcohol-dependence leads to major cerebral damage (McIntosh and Chick, 2004; Harper, 2007), particularly affecting white matter (Brooks, 2000; Oscar-Berman and Marinkovic, 2003), but also sub-cortical [e.g., amygdala (Cowen et al., 2004; Fein et al., 2006), insula, thalamus and cerebellum (Szabo et al., 2004; De Bellis et al., 2005)] and cortical [mainly temporal and frontal lobes (Kril et al., 1997; Harper and Matsumoto, 2005; Chanraud et al., 2007)] areas. These brain impairments correlate with the lifetime dose of ethanol consumed (Nicolás et al., 1997). At a functional level, many studies have explored the behavioral correlates of these cerebral effects, and have repeatedly shown impaired performance in a large range of cognitive abilities, ranging from perceptual (e.g., Blusewicz et al., 1977; Spitzer and Ventry, 1980; Spitzer, 1981) and attentional (e.g., Smith and Oscar-Berman, 1992; Sullivan et al., 1993; Noël et al., 2001) abilities to memory and executive functions (e.g., Bechara et al., 2001; Oscar-Berman et al., 2004; Flannery et al., 2007; Pitel et al., 2007). Nevertheless, in contrast with this extensive exploration of the consequences of alcohol-dependence on cognition, the evaluation of emotional abilities has long been neglected in this pathology.

### The emotional deficit

Emotional states clearly have a major influence on most aspects of our lives, as emotions plays a role in every human's decisions, motivations or social interactions. Moreover it has been repeatedly shown that affective disturbances (e.g., impaired ability to identify or regulate one's own emotional states or to decode other persons' emotions) constitute a central characteristic of most mental diseases from a clinical point of view. Despite this obvious importance of emotions in psychiatry, the interest for experimental exploration of affective impairments in alcohol-dependence only rose during the last decade. While this field is still in its infancy, it already clearly appears that alcohol-dependence is associated with major impairments in a wide range of emotional abilities. Several recent research projects identified an impaired performance in various emotional functions among alcohol-dependent individuals, notably for alexithymia (Taieb et al., 2002; Uzun et al., 2003), emotional intelligence (Riley and Schutte, 2003; Szczepanska et al., 2004; Cordovil de Susa Uva et al., 2010) and empathy (Martinotti et al., 2009; Maurage et al., 2011a). More centrally for the present purpose, a deficit has also been consistently observed for the decoding of the emotions expressed by faces (Oscar-Berman et al., 1990; Frigerio et al., 2002; Clark et al., 2007; Marinkovic et al., 2009; Maurage et al., 2009a, 2011b) and voices (Monnot et al., 2001, 2002; Uekermann et al., 2005). Recently detoxified alcohol-dependent individuals globally over-estimate the intensity of the emotions conveyed by visual and auditory stimuli, have an erroneous interpretation of emotions and are not aware of their deficit (Philippot et al., 1999; Kornreich et al., 2001, 2002). While several contradictory results have emerged, describing a preserved decoding of visual (Uekermann and Daum, 2008) or auditory (Oscar-Berman et al., 1990) stimulations, this emotional decoding deficit is now strongly established as it has been replicated in a wide variety of paradigms and stimulus sets (e.g., morphed or ambiguous faces, Frigerio et al., 2002), and among individuals with various abstinence durations (Townshend and Duka, 2003; Montagne et al., 2006; Foisy et al., 2007a). The causal link between alcohol-dependence and emotional impairment is still unclear as no longitudinal study has up to now directly explored this question, but individuals presenting a high risk of developing alcohol-dependence (i.e., children of alcohol-dependent patients) have strong emotional disturbances, and notably (1) altered activations of the brain areas involved in the elicitation and decoding of emotional feelings, particularly the amygdala (Glahn et al., 2007), (2) reduced social skills abilities and inefficient emotional coping strategies, as explored by self-report questionnaires (Segrin and Menees, 1996), and (3) higher frequency of psychopathological states known to have a strong effect on mood and emotional states, like depression or anxiety (Sher et al., 1991). It can thus be postulated that these emotional deficits might at least partly precede the appearance of alcohol-dependence. Moreover, while initially described for negative emotions, this impairment has been shown to be generalized to positive affective states if more complex emotional stimuli are used: when more various emotional and mental states are involved in the decoding task, alcohol-dependence leads to a deficit for negative and positive emotions (but not for neutral mental states), as recently shown in a study (Maurage et al., 2011b) exploring the performance of alcohol-dependent individuals at the “Reading the Mind in the Eyes” test (Baron-Cohen et al., 2001). It should also be underlined that this emotional decoding deficit appears specific for emotional features and cannot be fully explained by the more general cerebral and cognitive deficits observed in alcohol-dependence. Indeed, it has been shown that, when emotional decoding is compared with other tasks of similar complexity involving the identification of facial features (e.g., age, gender, or race detection tasks), alcohol-dependent individuals present a global deficit for performance and reaction times in each task. Nevertheless, when the general cognitive impairment is controlled for (using a reaction time subtraction method), alcohol-dependence is no more associated with an alteration for non-emotional face processing tasks but the emotional decoding deficit persists, suggesting that this deficit is specific and not only due to general cognitive alterations (Foisy et al., 2007b; Maurage et al., 2008a). To sum up, it is now clearly recognized that alcohol-dependence is associated with a general impairment for the decoding of the emotional content of stimulations, which is present for faces and voices, but also for other affective stimuli like music (Kornreich et al., 2013) or body postures (Maurage et al., 2009a).

## A more general deficit affecting social cognition

Importantly, it has been suggested that these emotional decoding impairments might influence social interactions and participate in the maintenance of the pathological state (Walitzer and Dearing, 2006). Indeed, as the development and preservation of adapted social communication is largely based on the ability to correctly express one's own emotional states and to accurately perceive (and react to) those expressed by others (Feldman et al., 1991), the emotional deficits might give rise to impaired interpersonal interactions and could increase the social problems frequently observed in alcohol-dependence. This assumption has not yet been directly tested in alcohol-dependence, but it has been shown that the intensity of emotional decoding alterations (specifically the over-estimation of negative emotions) is strongly correlated with the presence of interpersonal problems (as evaluated by the Inventory of Interpersonal Problems, Horowitz et al., 1988), and particularly with self-control difficulties in social contexts (Maurage et al., 2009a). While it has to be directly tested by prospective studies, this proposal of an involvement of emotional and interpersonal difficulties on the time course of alcohol-dependence recently received further empirical support by means of studies offering a specific exploration of the interpersonal abilities in alcohol-dependence. It has notably been shown that alcohol-dependent individuals have an impaired ability to understand humor (Uekermann et al., 2007), irony (Amenta et al., 2013) and mental states (Thoma et al., 2013), which are crucial abilities to correctly interact in interpersonal contexts. Moreover, alcohol-dependence is also associated with maladaptive self-beliefs in social context: alcohol-dependent individuals present an over-estimation of the social standards that have to be reached in order to be positively evaluated by others during social interactions, and their inability to actually reach these exaggerated social standards reduces their self-esteem and self-confidence when interacting with others (Maurage et al., 2013a). Finally, alcohol-dependence is also associated with a higher sensitivity to social rejection and ostracism (Maurage et al., 2012a), further underlining the role potentially played by emotional and interpersonal disturbances in this pathology.

Overall, better understanding the wide range of emotional disabilities related to alcohol-dependence (e.g., identifying, expressing and regulating one's own emotions, decoding and correctly reacting to others' emotions) is thus essential at fundamental level, but also for clinical practice, as they might have a role in the maintenance of this pathology, notably by hampering the development of satisfactory interpersonal links, thus potentially leading to social isolation and social stigma (Schomerus et al., 2011a,b). This social isolation could in turn reinforce the excessive alcohol consumption (used as a coping strategy to face isolation) and lead to a vicious circle (e.g., Carton et al., 1999). To sum up, the emotion decoding impairment in alcohol-dependence is now clearly identified and has a significant clinical importance, notably in view of its links with interpersonal problems. However, this deficit has up to now been exclusively explored using paradigms with low ecological validity, namely using only unimodal stimuli (faces or voices). It is thus unclear whether this deficit is maintained, reduced or increased in experimental designs that are closer to real life, specifically when crossmodal stimuli are used.

## Crossmodal emotional alterations in alcohol-dependence

As it has been described in the previous section, the affective deficits related to alcohol-dependence are now clearly established, particularly concerning the alterations in the decoding of emotional faces and voices. However, all these studies were based on unimodal explorations, i.e., on the separate presentations of faces and voices. Nevertheless, sensory events are not experienced in isolation in everyday life, as we are constantly immersed in a flow of multiple sensory cues carrying information from different sensory modalities. Crossmodal processing is thus the rule rather than the exception and this is particularly true for emotions, as the perception and production of emotional states are routinely based on several sensory aspects (e.g., emotional facial expressions and emotional prosody in crossmodal face-voice stimuli). Therefore, while constituting a valuable first exploration, the unimodal investigations of affective processing among alcohol-dependent individuals conducted up to now are insufficient to comprehend the complexity of emotion decoding processing in this population and should be extended to more ecological crossmodal designs. Following this observation, and on the basis of earlier crossmodal studies which explored the integration of emotional stimuli among healthy controls by means of electrophysiological (e.g., De Gelder et al., 1999; Pourtois et al., 2000, 2002; Chen et al., 2010) and neuroimaging techniques (e.g., Dolan et al., 2001; Ethofer et al., 2006a,b, 2013; Kreifelts et al., 2007, 2009, 2010; Müller et al., 2011), three studies were conducted in our research group to explore, for the first time to the best of our knowledge, the crossmodal emotional decoding in alcohol-dependent participants. Importantly, these studies joining behavioral, electrophysiological and neuroimaging techniques will illustrate the usefulness of a complementary approach combining cognitive psychology and neurosciences approaches to determine the modification of audio-visual emotional decoding in alcohol-dependence. It should also be noted that these three studies are focused on the comparison between recently detoxified alcohol-dependent participants (i.e., individuals diagnosed with alcohol-dependence according to DSM-IV criteria and recruited during their third week of treatment in a detoxification center) and healthy controls paired for age, gender and education. Moreover, alcohol-dependent participants had abstained from alcohol for at least 2 weeks before the experiment took place, thus excluding any potential influence of acute alcohol intoxication on the results observed. Finally, in order to ensure that the emotional decoding deficits were indeed associated with alcohol consumption and not with biasing variables, several control measures were conducted: (1) participants had normal or corrected-to-normal visual and auditory abilities; (2) major medical problems, neurological disease (including epilepsy) and other psychiatric diagnoses (as assessed by an exhaustive psychiatric examination), including polysubstance abuse, constituted exclusion criteria in both groups; (3) subclinical psychiatric comorbidities (particularly depression and anxiety) were controlled for by means of questionnaires [i.e., Beck Depression Inventory (Beck and Steer, 1987) for depression, State-Trait Anxiety Inventory (Spielberger et al., 1983) for anxiety]. These measures were entered as covariables in our statistical analyses to control for the influence of these subclinical psychiatric comorbidities.

### Do alcohol-dependent individuals present an emotional crossmodal facilitation effect?

**Figure 1:**
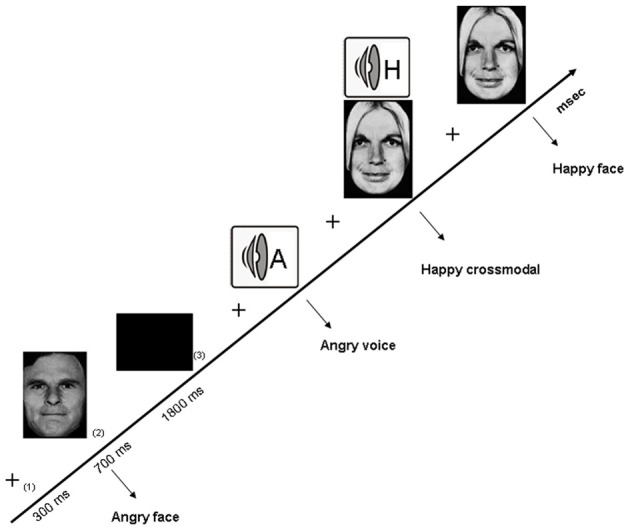
**Illustration of the experimental design related to the crossmodal facilitation experiment, with the successive arrival of (1) a fixation cross, (2) the stimulus, and (3) an inter-stimuli black screen**.The three categories of stimuli are illustrated, namely visual (angry face), auditory (angry voice) and crossmodal (happy face-voice).

**Figure 2:**
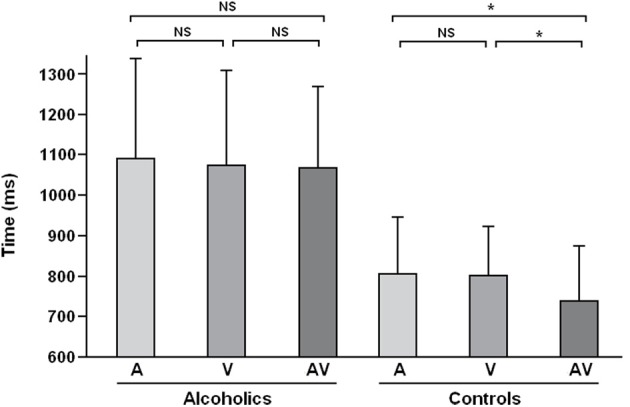
**Reaction times observed in the crossmodal facilitation experiment for the three modalities (A, Auditory; V, Visual; AV, Auditory-Visual) among alcohol-dependent participants (on the left) and controls (on the right)**. This panel shows that the facilitation effect (i.e., shorter reaction times for AV condition as compared to A and V ones) is present among controls but absent among alcohol-dependent participants (NS, Non-significant; ^*^p < 0.05). Adapted from Maurage et al. (2007a).

The first exploration of crossmodal emotional processing in alcohol-dependence (Maurage et al., 2007a) was based on the elicitation of a “crossmodal facilitation effect.” Most classical crossmodal paradigms [e.g., McGurk (McGurk and McDonald, 1976) and ventriloquist (e.g., Alais and Burr, 2004) effects] are based on inhibition effects (i.e., to a deteriorated performance in crossmodal conditions as compared to unimodal). Nevertheless, several more recent studies have developed paradigms leading to a facilitation effect (Calvert et al., 2001; Teder-Sälejärvi et al., 2002), in which congruent bimodal (audio-visual) stimulation leads to better performance (i.e., higher correct response rates and/or shorter reaction times) than unimodal ones. This facilitation effect is considered as the behavioral marker of successful crossmodal integration of stimuli from different modalities (Calvert et al., 2001), and the absence of this facilitation effect would conversely index impaired crossmodal integration. This study was thus based on a design eliciting a facilitation effect to evaluate the presence of this effect among alcohol-dependent individuals. More precisely, an emotion-detection task was used in which participants were presented with emotional facial expressions and voices [i.e., audiotapes enunciating a semantically neutral name with an emotional prosody, taken from a validated battery (Maurage et al., 2007b)] depicting anger or happiness (see Figure 1 for an illustration of the experimental design). Auditory and visual stimuli were presented separately (unimodal conditions) or simultaneously (crossmodal condition, with emotionally congruent face-voice pairs). Morphed faces were used (40–60% level morphs depicting 40% of happiness and 60% of anger, or conversely) in order to increase the perceptual difficulty of faces and to obtain similar levels of difficulty for vision and audition [as faces are classically processed more rapidly than voices (Ellis et al., 1997; Schweinberger et al., 1997; Joassin et al., 2004)], which is needed to obtain a facilitation effect. Participants (20 alcohol-dependent inpatients and 20 paired controls) had to decide as quickly as possible which emotion was displayed in the stimulus (anger or happiness). While alcohol-dependent individuals were not significantly impaired for crossmodal processing in terms of performance, reaction times results first showed that alcohol-dependent participants were globally slower than controls whatever the experimental condition, which is a classical visuo-motor slowing effect associated with alcohol-dependence (e.g., Fein et al., 1990). But the central outcome of this study is that, as illustrated in Figure 2, while control participants showed a clear facilitation effect (i.e., significantly shorter reaction times in the audio-visual condition than in the unimodal auditory and visual), alcohol-dependent individuals did not present this effect, as no differences were observed in this group according to the experimental condition. Alcohol-dependence is thus associated with an absence of crossmodal facilitation effect. As the facilitation effect is the behavioral marker of efficient crossmodal processing, these results show that alcohol-dependence is associated with impaired auditory-visual integration of complex ecological stimuli. These results constitute the first direct evidence of a crossmodal impairment in alcohol-dependence. Nevertheless, as no control neutral condition was used, it cannot be asserted that this deficit is specific for crossmodal emotional processing as it might also be present for non-emotional crossmodal integration. Moreover, this initial study focused on the behavioral description of the specific deficit for crossmodal processing in alcohol-dependence and did not allow us to explore the cerebral correlates of the deficit. Two complementary studies were thus performed to explore the brain impairments related to this audio-visual integration deficit.

### What are the brain correlates leading to the alteration of emotional crossmodal processes in alcohol-dependence?

#### At the neurophysiological level

**Figure 3:**
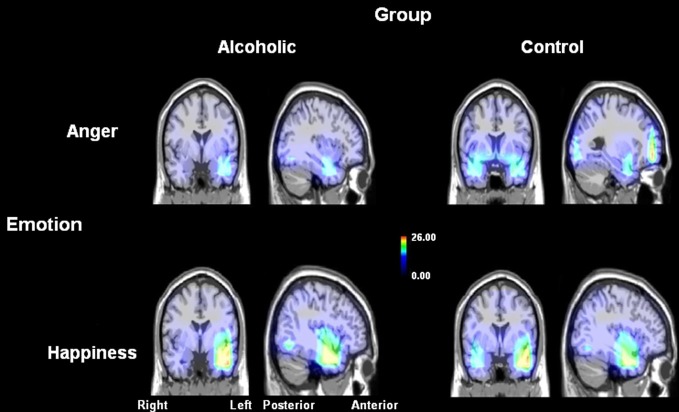
**Source reconstruction analysis (using swLORETA software) of the cerebral generators among alcohol-dependent participants (left) and controls (right), for anger (above) and happiness (below) electrophysiological subtraction [AV−(A+V)] waves**. Alcohol-dependent individuals exhibited a highly reduced frontal activation as compared to controls for anger stimulations. Adapted from Maurage et al. (2008b).

The second study (Maurage et al., 2008b) aimed at describing the brain alterations leading to audio-visual integration impairment, by means of event-related potentials (ERP). ERP record the brain's electrical activity during cognitive tasks with a high temporal resolution and allow us to identify the electrophysiological component associated with the onset of a dysfunction, and then to infer the cognitive stage related to this impairment (Rugg and Coles, 1995). ERP have been widely explored in alcohol-dependent participants during the last decades. The initial explorations (see Hansenne, 2006 for a review) repeatedly described a deficit (reduced amplitude and delayed latency) of the P3b, a long-lasting positive parietal deflection functionally associated with the decisional stage of processing (Polich, 2004, 2007). However, other studies described a deficit in earlier visual ERP components, like P100 (Ogura and Miyazato, 1991), N170 or N200 (Kathmann et al., 1996). These deficits for P100 (linked to early visual processing) and more importantly for N170 (linked to specific processing of faces) suggest that the impairment in alcohol-dependence begins before the decisional level (P3b), namely at the visuo-spatial level of cognitive processing (Maurage et al., 2007c). Therefore, ERP clearly help to identify the precise stage (e.g., perceptual, attentional or decisional) at which a behavioral deficit originates, and were used here to determine the initial cognitive stage responsible for the crossmodal integration impairment in alcohol-dependence, with the following central question: does the crossmodal deficit start at an early, perceptive stage or only at later processing steps? This second study also explored the potential differential deficit observed for positive (i.e., happiness) vs. negative (i.e., anger) emotions. An emotion detection task was performed by 15 alcohol-dependent participants and 15 paired controls, with visual and auditory stimuli (similar to those presented in the first study) presented separately or simultaneously for 700 ms. Participants had to decide as fast as possible whether the face, voice or face-voice stimulus was an angry, happy or neutral emotional expression. ERP were recorded using a 32 electrode cap to obtain, for each participant, several electrophysiological components of interest (P100, N170–N2, P3b) for each experimental condition (visual, auditory or audio-visual) and each emotion (anger, happiness, neutral). First, alcohol-dependent individuals were less accurate than control participants to identify the emotion depicted in face or voice presented alone, these data being in line with the repeated observation of a deficit in the emotion decoding in alcohol-dependence (e.g., Philippot et al., 1999; Townshend and Duka, 2003; Maurage et al., 2009a). Moreover, alcohol-dependence was associated with slower reaction times, which confirms earlier results (Beatty et al., 1996; Verma et al., 2006) showing a global visuo-motor slowing down in alcohol-dependence, independently of the task or stimuli used. More centrally, the results clearly confirmed the ERP deficits classically observed in alcohol-dependence, as alcohol-dependence was associated with reduced amplitude and delayed latency of the N170/N2 and P3b components for visual and auditory stimulations, thus confirming the ERP alterations repeatedly described in this pathological state (Hansenne, 2006). However, the main result of this study concerned the group differences for the cerebral activations specifically associated with crossmodal processing. Indeed, we used a classical subtraction technique (Teder-Sälejärvi et al., 2002; Joassin et al., 2004) to isolate the electrophysiological activities directly related to visuo-auditory integration, as the auditory (A) and visual (V) unimodal conditions were subtracted from the auditory-visual bimodal condition (AV) using the following formula: AV − [A + V]. Group comparisons on these specific crossmodal activities showed that alcohol-dependence leads to highly reduced brain activity during integrative processes. Moreover, this deficit is particularly present for anger stimuli, with a strong impairment starting as early as 100 ms after stimulus appearance (while the deficits for happiness and neutral stimuli only appeared after 200–300 ms and were far less marked). Finally, a source location analysis was conducted by means of standardized weighted low-resolution electromagnetic tomography (swLORETA, Palmero-Soler et al., 2007), a technique allowing to accurately reconstruct nearby current sources on the basis of the electroencephalographic data. This analysis showed that the anger crossmodal processing impairment is indexed by a reduction in frontal activity. These data, shown in Figure 3, thus complement the results obtained in the first study by showing (1) that early crossmodal processing of emotional stimulation is impaired in alcohol-dependence, particularly for anger, and (2) that this deficit is associated with a reduction of the electrophysiological activations specifically linked with integrative processes, particularly in frontal areas. However, it should be noted that the electrophysiological deficit found in the present study was not related to reaction times or accuracy alterations, as no specific deficit for emotional stimulations (as compared to neutral ones) was observed among alcohol-dependent individuals at behavioral level. This absence of specific emotional deficit at the behavioral level might be partly explained by the very low sensitivity of the task, which was very easy (leading to a ceiling effect in performance, with more than 90% of correct answers). As this combination between relatively preserved emotional processing at the behavioral level and strongly impaired emotional processing at the cerebral level has been repeatedly observed in electrophysiological and neuroimaging studies in alcohol-dependence (e.g., Maurage et al., 2013b), a potential complementary explanation is that alcohol-dependent individuals might develop alternative strategies to compensate for their deficit. For example, in the present study, they might focus on one sensory modality to compensate for their crossmodal deficit, which might partly hide their emotional decoding deficit. In line with this, it can not be totally excluded that part of the electrophysiological alterations observed here in alcohol-dependent individuals as compared to controls might be due to the use of these alternative strategies. This proposal of the use of alternative strategies to compensate for a deficit in easy tasks should nevertheless be confirmed by specific studies varying the difficulty of the task and the possibility to use these alternative strategies. Moreover, due to their low spatial resolution, ERP are not able to precisely localize the brain areas involved in this integration deficit. Therefore, these results had to be confirmed and complemented by the use of neuroanatomical techniques, which was the central objective of the third study.

#### At the anatomical level

This third study (Maurage et al., 2013b) aimed at precisely locating the cerebral regions responsible for impaired crossmodal processes in alcohol-dependence, by means of functional magnetic resonance imaging (fMRI). One the one hand, alcohol-dependence is known to be associated with major cerebral consequences, particularly in white matter, limbic, temporal and frontal areas. On the other hand, the emotional impairments presented by alcohol-dependent individuals are also well documented, particularly for the decoding of visual or auditory stimulation. Nevertheless, these cerebral and emotional alterations have traditionally been explored separately, and very little is known about the cerebral correlates of emotional impairments in alcohol-dependence. To our knowledge, only a few studies have specifically focused on this topic, comparing the brain activations of recently detoxified alcohol-dependent participants with that of controls during the presentation of emotional scenes (Heinz et al., 2007) or emotional facial expressions (Salloum et al., 2007; Marinkovic et al., 2009). These results show that alcohol-dependence is associated with reduced activity in a wide range of brain areas during the processing of emotional stimuli, encompassing frontal, parietal and temporal regions which are not specifically involved in emotion processing. More precisely, the most statistically significant activity reductions were shown in regions playing a role in emotional processing, particularly the inferior frontal gyrus, anterior cingulate cortex and limbic structures (particularly the amygdala and hippocampus). A more recent study (Schulte et al., 2010) also suggested that alcohol-dependence is associated with white matter abnormalities, thus leading to disconnections between brain areas, and mainly between cortical and limbic structures. As the cortico-limbic connections are central for the processing and interpretation of emotional signals, this white matter deficit could play a major role in the affective disorders observed in alcohol-dependence. Nevertheless, these studies were exclusively based on the presentation of visual emotional stimuli, and the brain correlates of auditory or audio-visual emotional processing remains unexplored.

### Alcohol-dependence leads to serious crossmodal alterations

**Figure 4:**
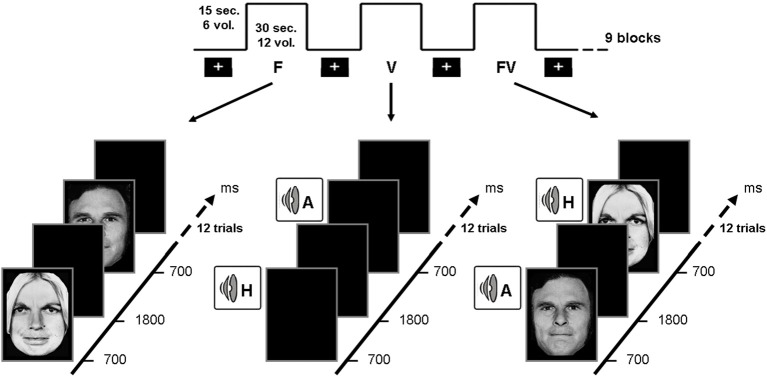
**Illustration of the experimental design related to the neuroimaging study, showing the three experimental conditions, namely visual (left), auditory (center) and crossmodal (right) stimulations**. Crossmodal stimulations were always congruent (i.e., depicting the same emotion in face and voice).

**Figure 5:**
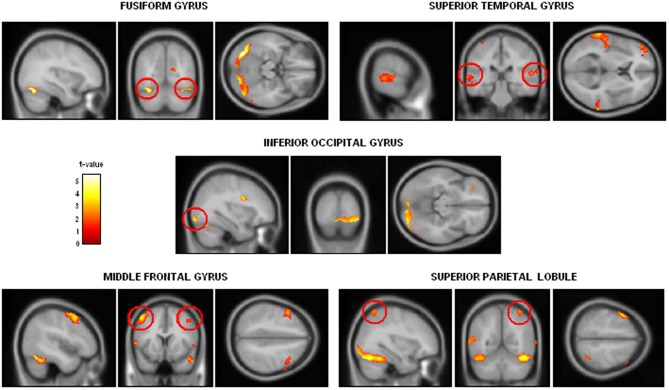
**Group comparison showing the cerebral regions presenting significantly reduced activations among alcohol-dependent participants when compared to controls for the [AV − [A + V]] contrast in the neuroimaging study (*p* < 0.05 corrected for multiple comparisons at cluster size)**. Adapted from Maurage et al. (2013b).

On the basis of the two studies presented above and of earlier results suggesting that brain areas dedicated to emotional processing are impaired or disconnected in alcohol-dependence, an fMRI study was conducted to explore the brain correlates of crossmodal emotional processing among alcohol-dependent participants. More precisely, an emotion detection task (illustrated in Figure 4) was administered to 12 alcohol-dependent participants and 12 paired controls while their brain activity was recorded using fMRI. The stimuli and task were identical to those presented in the first study, with a binary emotional decision (anger-happiness) on unimodal (morphed face or voice) or crossmodal (morphed face and voice presented simultaneously) stimulation. Brain activations during unimodal and crossmodal conditions were first computed (by subtracting the activations observed during a rest period without stimulation from those activations), and then the classical AV − [A + V] comparison was performed to isolate regions specifically involved in the integration of emotional faces and voices in both groups. It should first be underlined that alcohol-dependent individuals, while showing reduced unimodal activations (particularly in the middle frontal gyrus) presented a globally preserved pattern of cerebral activations during the separate processing of faces and voices. However, the central result of this study concerned crossmodal activations and the activations of the specific brain areas related to the integration of audio-visual stimulation. In the control group, the AV − [A + V] subtraction distinguished two categories of activations: on the one hand, several activations were found in unimodal regions (i.e., superior temporal gyrus for voices and fusiform gyrus for faces), showing that crossmodal stimulations provoke an enhanced activation in cerebral regions specialized in visual or auditory processing, which has been repeatedly observed among healthy participants (e.g., Calvert et al., 1999; Ghazanfar et al., 2005). On the other hand and more importantly, specific multimodal regions were revealed by the subtraction, namely middle frontal gyrus, superior parietal lobule and superior parietal gyrus. This is in line with earlier studies (e.g., Joassin et al., 2011a,b) showing that these brain regions are specifically activated in crossmodal conditions, as they receive multiple inputs from modality-specific regions and integrate them into a unitary and coherent representation of the environment (Rämä and Courtney, 2005; Bernstein et al., 2008). In the alcohol-dependent group however, the only significant activations for crossmodal stimulations were found in the unimodal regions cited above (mainly in the auditory regions), with a total absence of activations in the specific crossmodal areas. As further shown in the group comparison for crossmodal activities (Figure 5), it thus appears that alcohol-dependence is associated with a large and specific crossmodal deficit, indexed here by a lack of activation in the regions normally dedicated to the integration of inputs coming from different sensory modalities: alcohol-dependence is therefore associated with serious dysfunctions of the activation and connectivity between the cerebral regions involved in the multimodal perception of the social environment. Finally, psycho-physiological interactions (PPI) analyses allowed us to determine the functional connectivity between unimodal and crossmodal areas. Control participants presented a coherent connectivity pattern with on the one hand increased connectivity within unimodal regions (bilateral fusiform and superior temporal gyri), which confirms the enhanced unimodal connections in crossmodality (Kriegstein and Giraud, 2004), and on the other hand increased connectivity between unimodal and crossmodal areas (inferior occipital gyrus, middle frontal gyrus, superior parietal lobule), underlining the efficient functioning of the crossmodal cerebral network. Conversely, alcohol-dependent individuals did not present this pattern as unimodal areas were partially inter-connected but were not connected with crossmodal ones. As compared to controls, alcohol-dependent individuals showed highly reduced connectivity between unimodal and crossmodal areas, particularly with the middle frontal gyrus. These functional connectivity results thus suggest that the crossmodal deficit in alcohol-dependence could be partly due to disrupted connectivity within the crossmodal network, reducing the connections between unimodal and crossmodal areas. These data are preliminary and will have to be confirmed in future studies using larger groups and alternative experimental designs (Goebel and van Atteveldt, 2009; Love et al., 2011), notably by including neutral stimuli to explore the emotional specificity of these crossmodal alterations. However, they reinforce our earlier behavioral and electrophysiological results showing an emotional crossmodal processing deficit in alcohol-dependence, and offer the first description of the specific cerebral correlates of this impairment.

## Fundamental and clinical implications

The three studies presented above present a coherent pattern of results by describing similar specific audio-visual integration impairments in alcohol-dependence. Moreover, the use of different experimental methods and techniques provide complementary views of this impairment from behavioral, electrophysiological and neuroimaging data. Several implications can thus already be outlined on the basis of these results at experimental, fundamental and clinical levels, in order to lay the foundations for potential therapeutic interventions and future experimental investigation of these integrative processes.

First at the experimental level, the observation that the emotional decoding deficit in alcohol-dependence, widely described for unimodal stimulation, is increased in crossmodal stimulation, has shed new light on these earlier results and could influence future studies in the domain. Indeed, as crossmodal situations are omnipresent, our results suggest that earlier studies based on unimodal stimulation (and often using basic stimuli) underestimated the deficits in alcohol-dependence. This crossmodal impairment could also explain the hiatus between the relatively mild deficit frequently observed when presenting unimodal stimuli in experimental situations among alcohol-dependent subjects (e.g., Oscar-Berman et al., 1990; Beatty et al., 2000; Uekermann et al., 2005) and the obvious impairments observed in ecological situations, and notably in clinical observations. The present results could thus lead to a re-evaluation of earlier studies using unimodal stimuli, which probably underestimated the deficit present in real life situations. These results should also encourage future studies to use crossmodal stimulation in order to correctly evaluate various cognitive and emotional deficits in the processing of social stimuli. More generally, as emotional contexts in everyday life are most often characterized by the simultaneous perception of stimulations from different sensory modalities, our results argue for the development of more ecologically valid experimental designs, for example by means of video clips or virtual reality paradigms. Much progress has already been made in this direction for the evaluation of crossmodal processing among healthy participants (e.g., Vatakis and Spence, 2006; Barkhuysen et al., 2010; Petrini et al., 2010, 2011), and several studies have already used emotional crossmodal movies to explore face-voice integration (e.g., Kreifelts et al., 2010; Ethofer et al., 2013) but it has not been applied to clinical populations yet.

Second, from a more theoretical point of view, the development of experimental work on crossmodal processing in alcohol-dependence and other psychiatric states, could complement the results obtained among healthy participants and help to further renew the knowledge of crossmodal integration in general. Indeed, the exploration of impaired cognitive functions among clinical populations has traditionally been used, in neurology and neuropsychology, in order to add to those observations made among healthy individuals and to give a more exhaustive description of normal functioning. For instance, studies conducted among patients with cerebral lesions provided the description of double dissociations in memory or attentional systems, thus refining the theoretical models proposed for these systems (e.g., Listerud et al., 2009; Cohn et al., 2010; Barbeau et al., 2011). Specifically for the present topic, our fMRI results, showing that the crossmodal integration impairment in alcohol-dependence is associated with reduced activity in middle frontal gyrus, superior parietal lobule and superior parietal gyrus, confirms that these regions are necessary for a correct integration between faces and voices and thus reinforce the results obtained among healthy participants. This first step underlines the fact that exploring impaired crossmodal processing can offer promising perspectives, notably a better understanding of normal integration functioning.

Third, at the clinical level, the present results clearly confirm earlier data suggesting that emotional impairments (i.e., impaired ability to regulate one's own emotions or to correctly interpret others' emotional states) are a critical deficit in alcohol-dependence. The crossmodal paradigms used highlight that the impairments are more intense when experimental designs are closer to real life emotional situations. It is therefore obvious that emotional perturbations are involved in alcohol-dependence and should thus be considered in clinical settings, notably because they significantly contribute to relapse after detoxification,. Indeed, it has been shown by self-report questionnaires that more than 40% of alcohol-dependent patients identified the presence of emotional disturbances (e.g., persistent negative emotions, depression, anxiety) as being the most important causal factor explaining their relapse (Zywiak et al., 2003). Surprisingly, the experimental studies and theoretical models proposing therapeutic programs for alcohol-dependent individuals have up to now mainly focused on cognitive and behavioral aspects (e.g., development of coping strategies, motivation to change, DiClemente et al., 2003), and the emotional variables have been neglected. However, more recent models of addictions, and particularly the dual-process models (Wiers et al., 2007, 2013; Noël et al., 2010) offered a central position to emotional variables by considering that the addictive states are mostly due to an imbalance between “affective” (involved in automatic-impulsive behaviors) and “reflective” (involved in controlled-deliberate behaviors) systems. The present data, together with earlier results describing emotional alterations among alcohol-dependent individuals, further underline the importance of this affective system and encourage the development of therapeutic approaches focused on the rehabilitation of emotional abilities. Therapeutic programs have recently been developed to improve emotional decoding abilities among clinical populations by specifically training facial expression decoding (e.g., Face Tales Program, Philippot and Power, 2010). Applying them to alcohol-dependence, in complement with the classical rehabilitation programs including psychiatric and psychological therapy, might reduce the relapse rate after detoxification. Future development of these therapeutic proposals should include not only visual emotional stimuli, but also auditory and crossmodal stimulation, in order to develop more realistic emotion decoding rehabilitation programs. More globally, therapeutic interventions could also be improved through communication re-education programs in alcohol-dependence, focusing on crossmodal processing of the expression and identification of emotions in social settings.

Finally, in line with this clinical perspective, the specific crossmodal deficit for anger stimuli described in our electrophysiological study makes particular sense at the therapeutic level. Indeed, many clinical studies (e.g., Bartek et al., 1999; Karno and Longabaugh, 2005) have underlined that alcohol-dependent individuals have considerable difficulties to manage their anger, leading to aggressive behaviors and impulsivity in interpersonal relations. While the links between the regulation one's own anger and the ability to decode the anger expressed by others have not been directly explored yet, these two capacities might be simultaneously impaired in alcohol-dependence and sum up to increase interpersonal problems. However, although some studies have suggested that alcohol-dependence is associated with a relatively specific deficit in anger emotional facial expression decoding (e.g., Philippot et al., 1999), other studies have not replicated these results (Foisy, 2005; Uekermann et al., 2005) and this deficit has not been described for other stimuli (notably auditory prosody). This discrepancy between an obvious clinical deficit and contrasting experimental results could be explained by the fact that previous studies used only unimodal stimuli (mainly emotional facial expressions). These stimuli are artificial because in everyday social interactions, multimodal stimuli, and mainly auditory-visual stimuli, are much more common. Using more ecologic stimuli, our study established, at the electrophysiological level, the specific crossmodal deficit for anger in alcohol-dependence that has been repeatedly observed at clinical level. The development of future therapeutic programs should thus particularly emphasize the need to take into account this particular deficit for anger expression and decoding among alcohol-dependent individuals.

## Perspectives for future research

As outlined above, our studies have to be considered as a very initial exploratory step in the examination of emotional crossmodal processing among clinical populations. Indeed, these studies focused on a specific clinical population and used a small subset of the possible emotional stimuli and sensory modalities. Nevertheless, when combined with the few previous data sets obtained among other psychiatric populations, these results constitute a reliable and promising basis for the development of an ambitious research program aiming at determining the behavioral and cerebral correlates of impaired crossmodal integration in psychiatry, and finally leading to strong fundamental propositions as well as clinical applications. Before ending this paper, four main directions that should be developed in future research will be proposed, each focusing on the extension of previous results and proposing a diversification of the emotional stimuli used, sensory modalities included and psychiatric populations explored.

### Promoting the use of more emotional expressions

A main limitation of the results presented above is that they only considered a very low number of emotional states (i.e., happy, angry, neutral). A central direction for future research will be to diversify the emotional stimulation used to determine the potential differential deficits associated with different emotional states in alcohol-dependence. It can indeed be hypothesized that alcohol-dependent individuals' emotional crossmodal deficit will vary according to stimulus valence. Our ERP results suggested a specific deficit for anger as compared to happy and neutral stimuli. This specific deficit makes sense at the clinical level and could lead to the development of innovative therapeutic programs. Nevertheless, it is not clear whether this impairment is really limited to anger or is more general, as it could for example be present for every negative emotion. It is thus necessary to develop crossmodal experimental paradigms that explore a broader set of emotions, and particularly of negative ones (e.g., disgust, fear, sadness) to confirm our results and separate the hypotheses of an anger-specific deficit vs. a general deficit for negative emotions. It has also been suggested (e.g., Philippot et al., 1999; Maurage et al., 2008c) that alcohol-dependence could be associated with a particular deficit for decoding and correctly reacting to emotional states which have a high interpersonal value, and particularly which are associated with a social evaluation aspect or moral judgment (e.g., anger, disgust, contempt, e.g., Hutcherson and Gross, 2011), as compared to emotions which express more self-focused feelings (e.g., fear, sadness). Crossmodal paradigms, due to their high ecological validity, could be very useful in exploring these hypothetical propositions on the differential deficit between emotions presenting high vs. limited social value in alcohol-dependence. A recent study (Lambrecht et al., 2012) has performed this differential exploration between others-oriented and self-oriented emotions among healthy controls, and this experimental approach might be applied in alcohol-dependence to explore the impact of interpersonal value on emotional decoding. More generally, future studies focusing on integration processes in alcohol-dependence should also go beyond the exploration of classical emotion decoding. Indeed, emotional abilities are not limited to this basic emotion decoding as daily life forces us to identify and correctly react to far more various and subtle emotional signals. More complex affective abilities (e.g., empathy, emotional intelligence) are thus also needed to develop and maintain satisfactory interpersonal relations, and several studies have recently shown that alcohol-dependence is associated with impairments for these abilities (e.g., Martinotti et al., 2009; Maurage et al., 2011a,b). These results, in line with recent studies exploring more complex emotional states among healthy people (e.g., Basile et al., 2011; Wagner et al., 2011), underline the need to go further than conventional emotion labels and to use more subtle and ecological paradigms in order to develop a better understanding of emotional impairments in alcohol-dependence. Nevertheless, these studies were conducted by means of unimodal visual stimuli, and thus have a low ecological validity. The use of crossmodal paradigms exploring complex emotional states and affective abilities will enrich these preliminary results by bringing experimental designs closer to daily situations and thus by offering a more valid description of alcohol related emotional and affective disorders. In this view, it would also be particularly important to use dynamic emotional faces synchronized with dynamic voices, as static materials do not capture the liveliness and true form of the facial expressions that typically occur in day-to-day interactions (Sato et al., 2004), and as the emotion content of these “static” non-canonical stimuli is processed by mental strategies and neural events distinct from their more ecologically relevant dynamic counterparts (Kilts et al., 2003).

### Going beyond auditory and visual modalities

The crossmodal literature puts a strong emphasis on the integration between visual and auditory modalities. This focus is justified by the fact that vision and audition are by far the most dominant sensory modalities among human beings. Nevertheless, the near total absence of data concerning the other senses, and particularly the “chemical senses” (i.e., olfaction and taste) is surprising, as they play an underestimated but crucial role in the daily life of healthy as well as clinical populations (Schiffman, 1997). Indeed, olfactory and gustatory stimulation can also carry a strong emotional valence (e.g., Winston et al., 2005; Greimel et al., 2006; Shepherd, 2006), and exploring the integration between these emotional stimulations and visual or auditory ones could constitute a promising perspective to develop and renew crossmodal integration knowledge. More specifically, olfaction has been shown to play a crucial role in the development and maintenance of alcohol-dependence (e.g., Kareken et al., 2004; Little et al., 2005), but olfactory processing has up to now been studied very little in this pathology. Recent studies by our group exploring the olfactory abilities associated with excessive alcohol consumption (Maurage et al., 2011c,d) confirmed earlier results (Rupp et al., 2003, 2004, 2006) showing impaired processing of olfactory stimulations in alcohol-dependence, and gave the first insights concerning the cerebral correlates of this deficit (by means of ERP measures). Interestingly, our results showed that olfactory impairments are highly correlated with executive function deficits, and specifically with confabulation problems. These results suggest that these two abilities could rely on the same brain structures (and particularly on the orbitofrontal cortex), and that olfaction measures could be useful to shed new light on the exploration of executive and emotional impairments in alcohol-dependence. This is in line with recent proposals suggesting that olfactory measures could be a reliable cognitive marker in psychiatric disorders (Turetsky et al., 2009; Rupp, 2010). It thus appears that olfaction research is currently becoming a blooming research field among clinical populations. But once again, all these explorations have up to now been limited to unimodal stimulation while in real life situations, olfactory stimulations most often occur in combination with stimulation coming from other sensory modalities. This is particularly true for emotional contexts, and crossmodal explorations combining several senses (beyond audition and vision) are thus urgently needed to develop this new field of research. Very few studies have explored the crossmodal integration of emotional olfactory stimulation, by focusing on the influence of olfactory cues on facial expression decoding (Leppänen and Hietanen, 2003; Seubert et al., 2010a,b). These preliminary results replicated the classical facilitation effect, thus confirming the presence of genuine olfactory-visual integration among healthy participants. Neuroimaging data have also suggested that, while some brain areas (e.g., middle frontal gyrus) could be activated for every crossmodal interaction, independent of the sensory modalities engaged, other structures (mostly the anterior insula) could be specialized for olfactory-visual integration (e.g., Gottfried and Dolan, 2003; Small, 2004). Finally, they showed that schizophrenic patients present an impairment of this olfactory-visual integration, particularly for negative emotional stimuli, which suggests that crossmodal impairments among psychiatric populations could be independent of the sensory modalities involved. On the basis of these innovative explorations, future studies should thus explore, among healthy as well as clinical populations, the correlates of the crossmodal integration between the “chemical senses” and vision or audition. A more ambitious aim could be to go one step further toward ecological validity, by combining more than two sensorial modalities. Indeed, while our emotional experience is frequently based on the simultaneous perception of several sensory modalities, only bimodal stimulation paradigms have been proposed up to now. The recent technical advancements, and notably the growth of virtual reality, could lead to the development of experimental designs stimulating all the senses and thus open new perspectives for crossmodal processing explorations.

### Applying crossmodal paradigms to other psychiatric populations

The crossmodal studies presented in this chapter exclusively explored alcohol-dependence, and more specifically recently detoxified alcohol-dependent individuals. This population is of course only a specific part of the people presenting alcohol related problems, and more globally of the psychiatric patients. It thus appears important to underline the potential extension of these studies to other populations, in the field of alcohol abuse and dependence, but also in other psychiatric states, with the long term objective of developing a sound and integrative approach of crossmodal processing in clinical populations. Concerning alcohol related problems the literature on cerebral, cognitive and emotional impairments associated with alcohol consumption has classically been focused on long-term alcohol-dependence (namely on the exploration of the impairments due to long term chronic excessive alcohol consumption). Nevertheless, a new field of research has risen during the last decades, aiming at exploring the roots of alcohol addiction, namely the appearance and chronification of the deficits during the development of alcohol-dependence. On the one hand, many studies have been conducted among populations at high risk of becoming alcohol-dependent, mainly among children of alcohol-dependent individuals (Van der Stelt et al., 1994; Lieberman, 2000; Porjesz et al., 2005). These studies have suggested that several cerebral and cognitive impairments could be present before the development of alcohol-dependence, and thus be a causal factor rather than a consequence of excessive alcohol consumption. Our intention is not to go into the details of this important literature, but to underline that these explorations were once again exclusively based on unimodal stimulation. Crossmodal studies among children of alcohol-dependent individuals (notably for emotional abilities, which are still unexplored in this population) could thus give a more ecological and valid exploration of the deficits that are present before the development of alcohol-dependence. On the other hand and more recently, several projects have been conducted concerning the consequences of binge drinking (i.e., the excessive but episodic alcohol consumption, typically observed among adolescents and young adults and considered to be an “entrance door” toward alcohol-dependence, e.g., McCarty et al., 2004; Enoch, 2006). Recent studies have shown that binge drinking leads to cognitive effects (e.g., Giancola, 2002; Townshend and Duka, 2005; Zeigler et al., 2005), and we recently extended these results by suggesting that binge drinking habits rapidly lead to impaired processing of emotional auditory stimulation, and that this alcohol consumption pattern is particularly deleterious for brain functioning (Maurage et al., 2009b, 2012b). Nevertheless, it is still unknown whether these deficits are modified or not when several stimulations are presented together, and crossmodal studies would thus help to extend and clarify these preliminary results. Concerning the exploration of emotional crossmodal processing in psychiatry, it is surprising to notice that very few studies have been conducted among these clinical populations. Many projects have been proposed in recent years in order to explore the visual or auditory decoding of emotional stimulations among a wide variety of psychiatric states, like depression, autism, anxiety, anorexia nervosa and drug addiction (e.g., Mejias et al., 2005; Mendlewicz et al., 2005; Rossignol et al., 2005; Bhatara et al., 2010), but emotional crossmodal paradigms have only been used in a very limited number of these pathological states. Several studies (De Gelder et al., 2005; De Jong et al., 2009; Pearl et al., 2009; Szycik et al., 2009) have explored the integration of emotional stimulation among schizophrenic patients and consistently described emotional crossmodal deficits in this psychiatric state, notably indexed by reduced audio-visual integration ability and by a vision-audition imbalance (i.e., a dominance of the visual stimulation on the auditory one reducing crossmodal performance). These results, together with those obtained in alcohol-dependence, show that crossmodal processing impairments constitute a crucial aspect of psychiatric states, and should thus encourage the development of emotional crossmodal research among other psychiatric states. This is particularly true among populations which are known to present unimodal emotional decoding deficits, in order to answer the following central question: How does crossmodal integration occur when unimodal outputs are impaired? In other words, do some psychiatric populations manage to compensate their deficit in the processing of unimodal emotional stimuli by taking profit of the simultaneous presentation of two stimulations, or are all psychiatric states associated with increased processing impairments in crossmodal situations, as it has been observed in alcohol-dependence and schizophrenia?

### Using crossmodal tasks in real clinical settings

The final objective of this research area is also to offer new ways to manage clinical interventions in real clinical settings. Some preliminary data with clear potential clinical application have already been gathered (Campanella et al., 2010, 2012). Indeed, there is a growing literature base demonstrating that, throughout the information processing stream, a number of early and late neuroelectric features appear to be anomalous in various psychiatric populations. In all of these studies, the primary and most commonly reported finding has been P300 abnormalities (see Hansenne, 2006 for a review). P300 alterations have been highly important in the assessment of the pathophysiological mechanisms responsible for psychopathological states, as it is commonly acknowledged that a reduction of P300 amplitude is: (1) a state marker of depression, i.e., a biological marker that is altered during the disease but that stabilizes after clinical remission (Karaaslan et al., 2003); (2) a trait marker of schizophrenia, i.e., a biological parameter that is changed during and after the disease (Mathalon et al., 2000); and (3) a vulnerability marker of alcoholism, i.e., a biological variable that is altered before the emergence of the disease (high-risk children of alcoholic parents) (e.g., Hill et al., 1999). Such markers, if present, could be used to aid diagnosis, as prognostic elements, or to assist in choosing the most appropriate treatment for psychiatric disorders. They can also enhance our knowledge about the nature and the extent of cognitive damage, and offer deeper theoretical insights into both the aetiology and pathophysiology of the illness. Overall, such markers can improve early detection of illness, and, as such, facilitate more effective and targeted interventions (see Van der Stelt and Belger, 2007 for a review).

Nevertheless, the clinical sensitivity of the P300 has been hampered by the fact that its parameters (amplitude, latency) are diagnostically unspecific and not reliable enough to be useful for individual patients (Pogarell et al., 2007). Therefore, a current and important challenge for neurophysiologists is to discover novel and appropriate procedures to enhance the applicability and sensitivity of the P3b component in clinical settings. In this view, trying to increase P300 sensitivity, Campanella et al. (2010, 2012) have recently proposed a new variant of the classical oddball procedure using bimodal (visual-auditory) stimulations. The main idea was that, as bimodal face-voice associations require more “complex” interactions between unimodal (sensory) and multimodal (integrative) processes which work in parallel and influence each other, using a bimodal oddball design might enhance the sensitivity of the procedure by increasing the observable P300 differences evoked by “only” unimodal processes. To test this hypothesis, Campanella et al. (2010, 2012) compared two groups of participants: one group was healthy, and the other consisted of people displaying anxious and depressive tendencies. Both groups were submitted to unimodal (visual; auditory) and bimodal oddball tasks. Main results suggested that when the two groups of subjects differ in their subclinical level of comorbid anxiety and depression, unimodal visual and auditory oddball tasks may not allow us to detect this difference using P300 amplitude modulations, but a crossmodal task has greater power to detect even subclinical symptoms. Obviously, these results are preliminary and should be replicated on clinical populations: such experiments are currently underway in our laboratory, with the main aim to be able to index several steps of clinical severity in a pathological state. Moreover, it clearly appears that ERP data should not focus “only” on the P300 component: indeed, for instance, data have shown that (1) P300 deficits are correlated with previous “early” ERP alterations (e.g., Maurage et al., 2007c); and (2) the combination of different ERP components may be helpful to discriminate between different groups of patients. Price et al. (2006) compared and contrasted four electrophysiological endophenotypes -mismatch negativity, P50, P300, and antisaccades-, and analyzed their covariance on the basis of a single cohort of schizophrenic patients, family members and controls, tested with all paradigms. Data showed that the use of an electrophysiological battery has provided novel information on the characteristics of these features in schizophrenia and family member groups. In particular, it has highlighted the heterogeneity of electrophysiological features within these groups and how a combination of features could serve to minimize the impact of such heterogeneity. This outlines the urgent need in further studies to develop multisite guidelines to record a battery of electrophysiological measures that may be compared and used across studies.

## Conclusion

The exploration of crossmodal processing among healthy controls has now become an extensive research field: behavioral as well as cerebral correlates of the integration processes between sensory modalities have been precisely explored among animal and human populations, leading to comprehensive models on this topic. Nevertheless, this maturity of the knowledge concerning “normal” crossmodal processes is in total contradiction with what can be observed in clinical states. Indeed, as outlined above, very little has been done up to now to attempt to understand how these crossmodal processes are impaired among neurological and psychiatric populations, and this astonishing lack of interest has had a detrimental effect on the advances that can be made in this topic.

The main aim of the present paper was thus to underline the urgent need to explore the crossmodal integration abilities among these populations, as progressing in this direction could lead to central implications at clinical and fundamental levels. First, for clinical aspects, using crossmodal designs among clinical populations would lead to a better understanding of the impairments presented by inpatients in real life situations (and notably in emotional contexts). This would provide a more ecological exploration of the cognitive, cerebral and affective deficits in these populations, thus complementing and clarifying earlier results. This could also lead to the development of new therapeutic interventions, using crossmodal clinical settings to rehabilitate impaired abilities (e.g., by means of virtual reality). Second, for fundamental research, while the data obtained among clinical populations have traditionally constituted a strong method to improve the understanding of normal abilities in neuropsychology and neuroscience (with the well-known proposition that exploring an impaired system helps to understand its healthy functioning), this approach has received very little attention in crossmodal processing research. Developing the exploration of integration abilities among clinical populations could shed a new light on the several questions that are still unresolved in this research field.

By describing our research focusing on emotional crossmodal integration in alcohol-dependence, we have only presented here what can be considered as a modest first step toward a real and ambitious research program allowing to precisely describing the crossmodal processing abilities among psychiatric populations. We indeed believe that our work, together with the few studies conducted in schizophrenia, constitute seminal results that should be developed in the future. More specifically, studies to come should extend this exploration of crossmodal processing in at least four main ways, by diversifying the stimuli (i.e., using a wider range of emotional but also non emotional stimuli), the sensory modalities (particularly by including the “chemical senses” in the crossmodal designs), the populations explored (i.e., studying the crossmodal processes among other populations with substance-abuse, but also among other psychiatric and neurologic states), and by adapting experimental paradigms to real needs of current clinical settings. These proposals for future studies are just illustrations of the many prospects offered by this largely unexplored field. In short, everything is still to be done concerning crossmodal processing in psychiatric populations, and our hope is thus that the preliminary data described in the present chapter will open the door to fresh, diverse and complementary studies.

## Funding

The Authors are funded by the Belgian Fund for Scientific Research (F. N. R. S., Belgium), but this fund did not exert any editorial direction or censorship on any part of this article.

### Conflict of interest statement

The authors declare that the research was conducted in the absence of any commercial or financial relationships that could be construed as a potential conflict of interest.
